# Supplementary material for: Comparative multiomics analysis of cell physiological state after culture in a basket bioreactor
Source: Sci Rep. 2022 Nov 23;12:20161. doi: 10.1038/s41598-022-24687-4 (PMC9686226; doi:10.1038/s41598-022-24687-4)
Supplement: Supplementary file 1 — Supplementary Information 1. [file 41598_2022_24687_MOESM1_ESM.zip › raw data/Proteomic raw data/6.KEGGenrichment/EG.vs.CG/UP/pathway/src/04916.html]

map04916


- K02235, K02329, K02354, K02375, K02376, K02432, K02842

- Up regulated proteins


A0A0D9SCP4(Description:Uncharacterized protein;Gene:FZD1;log2FC:1.4940)

Close
